# Reasonability of Frequent Laboratory Analyses during Therapy with Nivolumab and Nivolumab+Ipilimumab in Patients with Advanced or Metastatic Renal Cell Carcinoma during the Phase 2 Clinical Trial TITAN-RCC

**DOI:** 10.3390/cancers16122287

**Published:** 2024-06-20

**Authors:** Klara Franke, Susan Foller, Michele Estephania Rosero Moreno, Nalyan Ali, Lutz Leistritz, Katharina Leucht, Marc-Oliver Grimm

**Affiliations:** 1 Department of Urology, Jena University Hospital, Friedrich-Schiller University, 07747 Jena, Germany; klara.franke@uni-jena.de (K.F.); susan.foller@med.uni-jena.de (S.F.); michele.roseromoreno@med.uni-jena.de (M.E.R.M.); nalyan.ali@med.uni-jena.de (N.A.); katharina.leucht@med.uni-jena.de (K.L.); 2 Comprehensive Cancer Center Central Germany (CCCG), 07743 Jena, Germany; lutz.leistritz@med.uni-jena.de; 3 Institute of Medical Statistics, Informatics and Data Science, Jena University Hospital, Friedrich-Schiller University, 07747 Jena, Germany

**Keywords:** immune-related adverse events, side effects, immune-checkpoint inhibitors, renal cell carcinoma, immune therapy, lab values

## Abstract

**Simple Summary:**

In this work, we evaluated the need for frequent laboratory assessments in patients with metastatic renal cell carcinoma treated with nivolumab and nivolumab+ipilimumab during the TITAN-RCC clinical trial. We analysed how often reached criteria for dose delay or permanent discontinuation of therapy would have been missed if the frequency of laboratory testing had been reduced, in order to avoid over-sampling patients and to optimise clinical workflow and staffing. Our study showed that if the frequency of laboratory tests had been reduced, reached dose delay criteria would hardly have been missed. This would have affected up to 1% (2/207) of patients. An exception was dose delay due to the elevation of lipase blood levels which would have been missed in up to 7% (15/207) of patients. However, these patients presented with symptoms and would have been identified based thereupon. Discontinuation criteria would have only been overlooked for lipase (2% [4/207] of patients) and amylase (0.5% [1/207] of patients). Again, these patients would have been identified, since only symptomatic patients would have needed to discontinue treatment due to amylase or lipase laboratory values. In conclusion, in asymptomatic patients in our setting, laboratory analyses do not seem to be necessary before every treatment application.

**Abstract:**

In clinical trials, laboratory values are assessed with high frequency. This can be stressful for patients, resource intensive, and difficult to implement, for example in office-based settings. In the prospective, multicentre phase 2 TITAN-RCC trial (NCT02917772), we investigated how many relevant changes in laboratory values would have been missed if laboratory values had been assessed less frequently. Patients with metastatic renal cell carcinoma (n = 207) received a response-based approach with nivolumab and nivolumab+ipilimumab boosts for non-response. We simulated that laboratory values were obtained before every second dose instead of every dose of the study drug(s). We assessed elevated leukocyte counts, alanine aminotransferase, aspartate aminotransferase, bilirubin, creatinine, amylase, lipase, and thyroid-stimulating hormone. Dose delay and discontinuation criteria were defined according to the study protocol. With the reduced frequency of laboratory analyses, dose delay criteria were rarely missed: in a maximum of <0.1% (3/4382) of assessments (1% [2/207] of patients) during nivolumab monotherapy and in a maximum of 0.2% (1/465) of assessments (1% [1/132] of patients) during nivolumab+ipilimumab boosts. An exception was lipase-related dose delay which would have been missed in 0.6% (25/4204) of assessments (7% [15/207] of patients) during nivolumab monotherapy and in 0.8% (4/480) of assessments (3% [4/134] of patients) during nivolumab+ipilimumab boosts, but would have required the presence of symptoms. Discontinuation criteria would have only been missed for amylase (<0.1% [1/3965] of assessments [0.5% (1/207) of patients] during nivolumab monotherapy, none during nivolumab+ipilimumab boosts) and lipase (0.1% [5/4204] of assessments [2% (4/207) of patients] during nivolumab monotherapy; 0.2% [1/480] of assessments [0.7% (1/134) of patients] during nivolumab+ipilimumab boosts). However, only symptomatic patients would have had to discontinue treatment due to amylase or lipase laboratory values. In conclusion, a reduced frequency of laboratory testing appears to be acceptable in asymptomatic patients with metastatic renal cell carcinoma treated with nivolumab or nivolumab+ipilimumab.

## 1. Introduction

Immune checkpoint inhibitors (ICI), either as mono- or combination therapies, have been established as therapeutic options for various cancer types [1], including metastatic renal cell carcinoma (mRCC) [2,3,4,5,6,7,8]. A commonly used combination approved for first-line treatment of patients with intermediate or poor risk mRCC combines the anti-PD-1 ICI nivolumab and the anti-CTLA-4 ICI ipilimumab [3].

In the TITAN-RCC study, patients with mRCC received a response-based approach with nivolumab and nivolumab+ipilimumab boosts for non-response [9]. ICI therapy is commonly associated with immune-related adverse events, mainly concerning skin, liver, colon, lungs, and the endocrine system [10,11,12]. In the protocol of TITAN-RCC, certain criteria were defined that would require a delayed dose application or even permanent discontinuation of study medication, many of those being based on laboratory values. This is in accordance with the up-to-date investigator brochure or, in the case of already approved medication, the official summary of product characteristics (SmPC) published by the European Medicines Agency (EMA) [13]. The study protocol includes the assessment of several laboratory values prior to each dose of the study drug(s), which may be used for decisions about continuation, dose delay, or discontinuation. Especially at sites participating in clinical trials, this is a standard procedure and may be implemented relatively easily due to available structures and capacities in the laboratories involved. In everyday clinical practice, however, approved therapies are also administered by resident physicians. When blood sampling is carried out in office-based settings, it usually takes longer for the results of laboratory value analyses to be available. Additionally, the procedures tie up time and personnel. For this reason, we have investigated whether, retrospectively, the high frequency of laboratory value analyses applied in TITAN-RCC would have actually been necessary with regard to detection of laboratory value alterations, determination of adverse events, and consequences for continuation of therapy.

## 2. Materials and Methods

### 2.1. Study Design and Participants

TITAN-RCC was a multicentre, single-arm, phase 2 trial that recruited patients at 28 sites across Europe (Austria, Belgium, Czech Republic, France, Germany, Italy, Spain, and the UK). The study design, and its inclusion and exclusion criteria have been reported previously [9]. In brief, eligible patients were adults (aged ≥18 years) with histologically confirmed advanced or metastatic renal cell carcinoma (RCC) with a clear cell component and intermediate or poor risk according to the International Metastatic Renal Cell Carcinoma Database Consortium (IMDC) [14]. Patients were either formerly untreated (first-line) or pre-treated with one previous systemic therapy (anti-angiogenic or temsirolimus; second-line). Measurable disease per Response Evaluation Criteria in Solid Tumours [15], a Karnofsky Performance Status score ≥ 70, and a tumour sample for analysis of PD-L1 expression were mandatory.

The trial was approved by all relevant national competent authorities and independent ethics committees, and conducted in compliance with the Declaration of Helsinki [16], Good Clinical Practice, and local regulatory requirements. All patients provided written informed consent before enrolment in the study. TITAN-RCC is registered with ClinicalTrials.gov, NCT02917772.

### 2.2. Procedures

**Figure 1:**
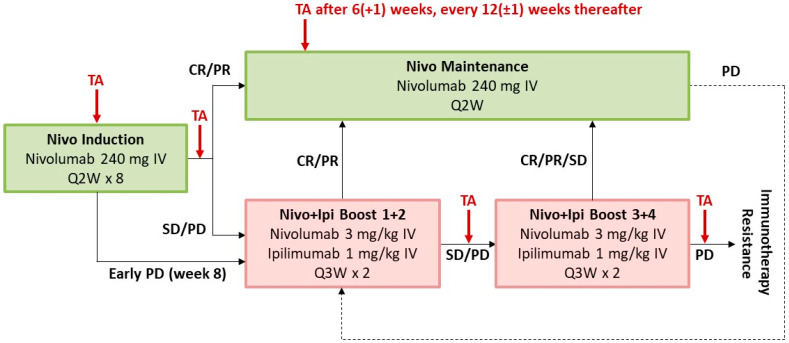
Study design. All response information is taken from time point response data. Nivo, nivolumab; Ipi, ipilimumab; IV, intravenous; Q2W/Q3W/Q4W, once every 2/3/4 weeks; SD, stable disease; PD, progressive disease; CR, complete response; PR, partial response; TA, tumour assessment.

The study procedures have been described in detail before [9]. The study scheme is displayed in Figure 1. All patients started treatment with induction nivolumab monotherapy (240 mg, intravenous) once every 2 weeks for 16 weeks. On early progressive disease (week 8) or non-response during tumour assessment (CT or MRI) at week 16, patients received either two or four doses of intravenous nivolumab (3 mg/kg) plus ipilimumab (1 mg/kg) boosts (once every 3 weeks), whilst responders continued with intravenous nivolumab (240 mg, once every 2 weeks). In case of progressive disease during the treatment, the responders could also receive two to four boost doses of nivolumab (3 mg/kg) plus ipilimumab (1 mg/kg) boosts. After two or four boost cycles with resulting complete response, partial response, or stable disease, the patients received intravenous nivolumab (240 mg, once every 2 weeks). Patients who had a progressive disease after four boost cycles were defined as immune therapy resistant. Tailored treatment was administered until immunotherapy resistance, unacceptable toxicity, or withdrawal of consent. After discontinuation, patients completed two follow-up visits 4 weeks after the last dose and 12 weeks thereafter and were subsequently monitored for survival every 3 months.

### 2.3. Laboratory Assessments

In TITAN-RCC, the following laboratory values were assessed at screening (within 14 days prior to registration), within 72 h prior to every dose of nivolumab induction monotherapy, nivolumab maintenance monotherapy, and nivolumab+ipilimumab combination therapy, as well as at both follow-up visits: differential blood count, erythrocytes, thrombocytes, haemoglobin, haematocrit, alanine aminotransferase (ALAT), aspartate aminotransferase (ASAT), alkaline phosphatase, bilirubin, serum urea, creatinine, calcium, albumin, magnesium, sodium, potassium, chloride, lactate dehydrogenase (LDH), glucose, amylase, lipase, thyroid-stimulating hormone (TSH), free triiodothyronine, and free thyroxine. For this work, we focussed on the laboratory parameters associated with common adverse events that can be diagnosed using laboratory parameters: leucocytes (activation of the immune system in general); ALAT, ASAT, bilirubin (immune-related hepatitis); creatinine (immune-related nephritis and renal dysfunction); amylase, lipase (pancreatic dysfunction), and TSH (hypothyroidism).

### 2.4. Outcomes

In our exploratory analyses, we assessed how often and at what time dose delay and discontinuation criteria were reached across the selected laboratory values. These criteria were defined and applied according to the protocol of TITAN-RCC based on CTCAE v4.0 [17]. As a control, we applied the respective criteria according to the current version of the SmPC for nivolumab published by the EMA (last updated 4 April 2024) [13], being also valid for the combination of nivolumab+ipilimumab and based on CTCAE v4.0. All applied criteria are summarised in Table 1. If the presence or absence of symptoms was included for a respective criterion, these were looked up in the patients’ records.

To determine the necessity of frequent assessments of the selected laboratory values, we simulated that blood analyses had been performed with reduced frequency. The simulated frequencies were defined for each of the different phases of the therapy: nivolumab induction monotherapy, nivolumab+ipilimumab boost, and nivolumab maintenance monotherapy. We simulated that laboratory values had been determined at the first and second visit of each of the three treatment phases, and at every second visit thereafter (Table 2, variant A). As a control, we furthermore simulated that laboratory values had been determined at the first visit of each of the three treatment phases, and at every second visit thereafter (variant B). Data reported in the main paper refer to variant A, as this is considered to be more relevant in practice (control of laboratory values after the first administration of medication). The data we obtained with variant B are shown in the supplement. For nivolumab+ipilimumab boost phases this meant that laboratory values would have been taken before the first, second, and fourth of four doses, and, as a control, before the first and third of four doses. We assumed that during follow-up visits no laboratory values were assessed.

We recorded relevant events that occurred during the visits that were simulated as being without laboratory testing. These relevant events were considered as overlooked. Relevant events were as follows: (i) elevation of laboratory values by at least one CTCAE grade, regardless of consequences for the continuation of therapy, (ii) reaching of dose delay criteria (according to the TITAN-RCC protocol or to the SmPC of nivolumab), and (iii) reaching of discontinuation criteria (according to the TITAN-RCC protocol or to the SmPC of nivolumab).

### 2.5. Statistical Analysis

Statistical calculations were performed with SPSS statistical software (version 28.0.0.0).

Demographics and baseline characteristics were summarised using descriptive statistics. Descriptive statistics were also applied to determine how often relevant events were overlooked across all of the selected laboratory values. Dose delay and discontinuation criteria that would have been overlooked in our setting were evaluated separately for nivolumab monotherapy (induction or maintenance) and for nivolumab+ipilimumab boosts. The differences between variants A and B on a per-patient level were assessed using McNemar’s test. Time-to-event distributions were estimated using Kaplan–Meier methodology. A log rank test was used for comparison of different simulations.

## 3. Results

### 3.1. Baseline Characteristics

The study baseline characteristics have been reported in detail before [9]. Between 28 October 2016 and 30 November 2018, 207 patients were enrolled, with 109 patients being first-line and 98 second-line. Of the patients, 147 (71%) were male and 60 (29%) were female. A total of 147 patients (71%) had intermediate risk according to IMDC and 51 (25%) had poor risk. Upon source data review, nine patients (4%) were identified who had favourable IMDC risk. IMDC risk factors which form the basis for determination of the risk groups are detailed in Table 3. In the overall population, median follow-up was 27.6 months (interquartile range [IQR] 10.5–34.8).

### 3.2. Rate and Time of Reaching of Dose Delay and Discontinuation Criteria

**Figure 2:**
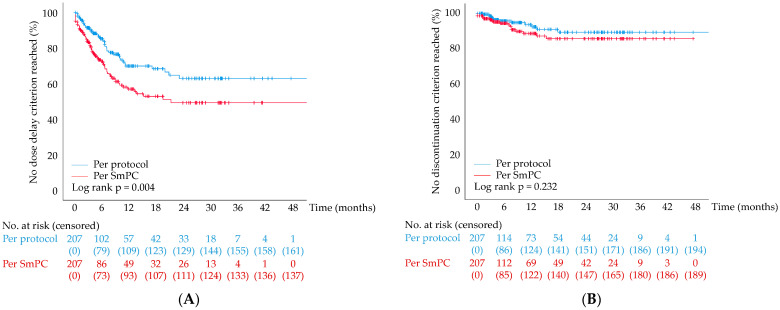
Reached dose delay and discontinuation criteria. (**A**) Patients without dose delay criterion reached as defined by the protocol of TITAN-RCC vs. as defined by the SmPC of nivolumab; (**B**) patients without discontinuation criterion reached as defined by the protocol of TITAN-RCC vs. as defined by the SmPC of nivolumab. All analyses are based on CTCAE v4.0. CTCAE, Common Terminology Criteria for Adverse Events; SmPC, summary of product characteristics/product information; NAR, number at risk.

During the course of the study, 46/207 patients (23.2%) met at least one dose delay criterion as defined according to the protocol of TITAN-RCC across all pre-selected laboratory values. When defining dose delay criteria per SmPC of nivolumab, this applied to 70/207 patients (34%). The distribution over time significantly differed depending on the definition applied (log rank *p* = 0.004, Figure 2A). For both, however, dose delay criteria were mainly reached during the first 12 months of therapy.

With regard to these laboratory values, discontinuation criteria were met in 12/207 patients (6%) (per protocol) and 18/207 patients (9%) (per SmPC), respectively. The distribution over time is displayed in Figure 2B (log rank *p* = 0.232).

### 3.3. Simulation of Reduced Frequency of Assessment of Laboratory Parameters

#### 3.3.1. Elevation of Laboratory Values by at Least One CTCAE Grade, Regardless of Consequences for the Continuation of Therapy

The laboratory values assessed within this article have been analysed between 4606 and 5487 times in 207 patients over the course of TITAN-RCC.

Increased levels of laboratory parameters by at least one CTCAE grade (based on CTCAE v4.0), irrespective of consequences for treatment continuation, would have been missed in from 18/207 patients (9%, bilirubin) to 80/207 patients (39%, creatinine). This corresponds to a total number of missed laboratory value elevations ranging from 25/5404 analyses (0.5%, bilirubin) to 192/5475 analyses (4%, creatinine) (Table 4). Applying the control simulation (variant B) did not produce significantly different results for any of the laboratory values (Supplementary Table S1). On a per-patient level, a median of 22% (IQR 17–22) of patients would have been affected.

#### 3.3.2. Reaching of Dose Delay or Discontinuation Criteria

During nivolumab monotherapy phases of TITAN-RCC, the assessed laboratory values have been analysed between 3965 and 4522 times. During the nivolumab+ipilimumab boost phase, they were evaluated between 465 and 518 times.

##### Criteria Defined According to the TITAN-RCC Protocol

For leukocytes, ALAT, ASAT, bilirubin, creatinine, and TSH, reached dose delay criteria were most frequently missed during nivolumab monotherapy for ASAT (2/4327 [<0.1%] lab value assessments) and during nivolumab+ipilimumab boost phases for bilirubin (1/510 [0.2%] assessments). For details see Table 5. Regarding amylase, reached dose delay criteria would have been missed in 2/3965 (<0.1%) assessments during nivolumab monotherapy. This would have affected 2/207 (1%) symptomatic patients. During nivolumab+ipilimumab boost phases, for amylase in 1/465 (0.2%) assessments a reached dose delay criterion would have been missed. The affected patient was symptomatic. As for lipase, reached dose delay criteria would have been missed in 25/4204 (0.6%) assessments (15/207 [7%] symptomatic patients affected) during nivolumab monotherapy. During the boost phases, reached dose delay criteria would have been missed in 4/480 (0.8%) assessments. A total of 4/134 (3%) patients would have been affected. All were symptomatic.

For leukocytes, bilirubin, creatinine, and TSH no discontinuation criteria were met at any laboratory assessment, neither during nivolumab monotherapy (induction or maintenance) nor during nivolumab+ipilimumab boost phases. Accordingly, no reaching of a respective discontinuation criterion would have been missed. ALAT- and ASAT-emergent discontinuation would have been required once over the course of the trial during nivolumab+ipilimumab boost. However, reaching of these discontinuation criteria would not have been missed. These data are mirrored on the per-patient level (Table 5). For amylase, in 1/3965 (<0.1%) assessments during nivolumab monotherapy a reached discontinuation criterion would have been missed. However, symptoms were required for the discontinuation criterion to be met. Criteria for lipase-derived discontinuation would have been missed in 5/4204 (0.1%) assessments during nivolumab monotherapy, and in 1/480 (0.2%) during nivolumab+ipilimumab boost phases. This affected 4/207 (2%) patients during nivolumab monotherapy and 1/134 (0.7%) patient during boost. Of note, these patients were symptomatic, a prerequisite for a lipase-emergent discontinuation criterion to be met.

Data derived from the control simulation (variant B) were comparable and not statistically different (Supplementary Table S2).

##### Criteria Defined According to the SmPC of Nivolumab

For leukocytes, ALAT, ASAT, bilirubin, amylase, and TSH, numbers of overlooked dose delay and discontinuation criteria marginally differed from those described before, when defining the respective criteria according to the TITAN-RCC protocol. For details see Table 6.

For creatinine, clearly more reached dose delay criteria would have been overlooked than when applying the criteria according to the TITAN-RCC protocol: 134/4511 times (3%, 19 patients affected) during nivolumab monotherapy, and 9/518 times (2%, six patients affected) during nivolumab+ipilimumab boost phases. No reached discontinuation criteria would have been overlooked.

Regarding lipase, considerably fewer dose delay criteria would have been overlooked than when applying the criteria defined according to the TITAN-RCC protocol: 7/4204 times (0.1%) during nivolumab monotherapy, and 2/480 times (0.4%) during nivolumab+ipilimumab boost phases. The criteria could only be met once per patient, since they were defined as *first* occurrence of CTCAE grade 3. A total of 3/7 (43%) patients affected during nivolumab monotherapy were asymptomatic. Missed dose delay criteria during the boost phases only occurred to symptomatic patients. In contrast, more patients reaching discontinuation criteria would have been overlooked during nivolumab monotherapy (14/4204 [0.3%] times, 7/207 [3%] patients affected) and nivolumab+ipilimumab boost (2/480 [0.4%] times, 2/134 [1%] patients affected). A total of 3/7 (43%) patients affected during nivolumab monotherapy showed neither symptoms nor had they previously shown increased lipase levels. 

Data derived from the control simulation (variant B) were comparable and not statistically different (Supplementary Table S3).

## 4. Discussion

In this work, we simulated a reduced frequency of laboratory testing as compared to the one actually implemented over the course of the phase 2 TITAN-RCC clinical trial in patients with intermediate or poor risk mRCC [9]. We assessed whether this would have been sufficient to detect laboratory changes, to identify adverse events, and to draw conclusions for treatment continuation.

In TITAN-RCC, the majority of patients already had laboratory values outside the normal range at baseline, before starting treatment with checkpoint inhibitors. This directly related to the study’s inclusion criteria. In line with the approved indication for nivolumab+ipilimumab in mRCC, the study population was limited to patients with intermediate and poor risk according to IMDC, which in turn is decisively influenced by haemoglobin, platelets/thrombocytes, neutrophils, and (corrected) calcium. Therefore, laboratory values outside the normal range were also to be expected during this study.

The choice of laboratory parameters analysed here was based on the common immunotherapy-related adverse events that can be diagnosed using laboratory values. These included immune-related hepatitis (ALAT, ASAT, bilirubin), immune-related nephritis and renal dysfunction (creatinine), pancreatic dysfunction (amylase, lipase), and hypothyroidism (TSH) [10,11,12,18,19].

When the frequency of laboratory testing was reduced, the number of missed CTCAE-grade elevations and the number of patients affected differed between the laboratory values analysed. However, the simulation of variants A or B (Supplementary Tables S1–S3) gave comparable, statistically non-significant results per laboratory value. For a median of 22% of the patients, an elevation of at least one CTCAE grade would have been missed. On a per-assessment level, rates of missed elevations were clearly lower, ranging from 0.5% (bilirubin) to 4% (lipase). It should be noted that an increase in CTCAE grade per se would not necessarily have indicated the need to discontinue or delay drug administration, e.g., an increase from CTCAE grade 0 to 1 or grade 1 to 2. Therefore, the analysis of missed criteria for dose delay and discontinuation was decisive.

Dose delay and discontinuation criteria were defined in the TITAN-RCC study protocol. They mainly depended on CTCAE grading (v4.0), taking into account a relative increase above the upper limit of normal and (for liver values and creatinine) baseline data. Dose delay and discontinuation criteria for amylase and lipase during TITAN-RCC required the presence of symptoms. However, ICIs represent relatively new therapeutics. In Europe, nivolumab was approved in 2016 [8,20] for second-line treatment of mRCC, and nivolumab+ipilimumab in 2019 for first-line treatment [3,21]. The first protocol version of TITAN-RCC was developed in 2016. Over time, experience with nivolumab and nivolumab+ipilimumab has increased and real-world data have improved knowledge on side effects and their management [22,23,24,25]. For this reason, the SmPCs have been and continue to be regularly updated. As a consequence, the criteria defined for TITAN-RCC do not necessarily apply to current clinical routines, so we additionally evaluated our data using the recommendations of the latest version of the SmPC for nivolumab as a control. It should be noted that the SmPC also explicitly refers to CTCAE v4.0, although version 5.0 [26] would be the most up to date. The criteria defined in the SmPC are not specific to a particular tumour type but apply to all tumour entities for which nivolumab is approved. Results significantly differed depending on the respective definition of dose delay and discontinuation criteria. This may be mainly due to different definitions of criteria for creatinine, lipase, and TSH. Using the SmPC, for creatinine and TSH a dose delay criterion was met from CTCAE grade 2 (instead of grade 3 using the TITAN-RCC protocol), resulting in more patients being affected. For creatinine, however, it is not clear to what extent elevated blood levels are associated with the underlying renal disease or with possible immune-related side effects, such as colitis leading to exsiccosis resulting in (pre-)renal impairment. Unlike other tumour types, patients with renal tumours often live with a solitary kidney and have limited kidney function. The requirement for a general dose delay from CTCAE grade 2 may be too strict for renal disease, and for mRCC the disease-specific criteria of the TITAN-RCC protocol may be more appropriate than the cross-entity criteria defined in the SmPC.

Another major difference between the definitions of dose delay and discontinuation criteria concerned lipase and amylase. The criteria defined in the SmPC (based on CTCAE v4.0) were more restrictive. First, in contrast to the protocol, only the *first occurrence* of CTCAE grade 3 resulted in dose delay, whereas *recurrent* CTCAE grade 3 required permanent discontinuation of therapy. Second, the recommendations for dose delay or discontinuation were independent of the presence of symptoms [13]. However, it should be recognised that the current version of CTCAE, v5.0 [26], includes symptoms in the grading assessment for amylase and lipase. For the few amylase-related events that occurred, the differences in the definition of the criteria between the TITAN-RCC protocol and the SmPC had little effect. In contrast, the results for lipase shifted towards fewer met (and missed) dose delay criteria and towards more met (and missed) discontinuation criteria when using the SmPC instead of the protocol criteria. We believe that the symptom-based management of elevated laboratory values according to the TITAN-RCC protocol is more appropriate in our setting. However, the SmPC of nivolumab may be most commonly consulted in practice when managing dose delay and discontinuation criteria. This could be seen as critical, as symptoms are not taken into account when deciding on amylase- or lipase-dependent (dis)continuation of therapy.

Events requiring dose delay or discontinuation occurred mostly during the first 12 months of therapy. After this period, it became increasingly unlikely that these would be missed when choosing the simulated lower frequency of laboratory assessments. Over the entire duration of the study, missed dose delay and discontinuation criteria were not very common in the simulated settings. During the nivolumab+ipilimumab boost phases, the rates of affected patients were lower than during nivolumab monotherapy. We believe that this is due to the lower total number of nivolumab+ipilimumab administrations compared to nivolumab monotherapy and the corresponding laboratory assessments.

Irrespective of the definition and variant used, discontinuation criteria were only missed for lipase, amylase, and (once, during control simulation, see supplement) for ALAT. However, if the criteria according to the protocol of TITAN-RCC were used, amylase- and lipase-based discontinuation required the presence of symptoms (e.g., nausea, abdominal pain, and fatigue) that would not have been missed.

In general, with a reduced frequency of laboratory assessments, dose delay criteria would have been missed more often than discontinuation criteria, regardless of the applied definitions and variants. For each laboratory parameter assessed, a dose delay criterion would have been missed at least once in our simulated scenarios. Most missed relevant events occurred only once per patient and laboratory value, with the exception of lipase and creatinine, for which in some patients dose delay or discontinuation criteria would have been missed repeatedly. If one decides to reduce the frequency of laboratory testing, it could be considered to re-increase the frequency for lipase and creatinine if the dose delay criteria were met at least once.

To date, in routine clinical practice, nivolumab+ipilimumab is approved for patients with intermediate or poor risk mRCC, starting with dual checkpoint inhibition with nivolumab (3 mg/kg) and ipilimumab (1 mg/kg) once every 3 weeks for four doses and followed by nivolumab monotherapy 240 mg once every 2 weeks or 480 mg once every 4 weeks. The phase 3 CheckMate-214 trial was pivotal for the combination therapy and introduced the dosing regimen of 240 mg of nivolumab every 2 weeks during the monotherapy phase [3,27]. The option of using a higher dose of nivolumab at a reduced frequency came up later. Accordingly, the TITAN-RCC protocol demanded a dose of 240 mg of nivolumab every 2 weeks during induction and maintenance monotherapy. Due to the COVID-19 pandemic and the fact that it had been approved by then, the frequency was reduced in some cases to 480 mg every 4 weeks. This was not taken into account in our analysis. We strictly adhered to the specified simulation(s) and considered every second laboratory assessment as non-existent, regardless of whether there were 2 or 4 weeks between nivolumab administrations. In clinical routine, close monitoring is often undertaken, in particular at the beginning of a therapy, as most side effects occur early in the course of treatment [28]. We found no evidence that more frequent laboratory assessments were needed, either at the beginning or during the course of treatment. Also, interim assessments in patients receiving nivolumab every 4 weeks at a dose of 480 mg do not appear to be necessary in asymptomatic patients, and our chosen frequency of laboratory assessments in combination with the patients’ clinic seemed to be sufficient.

## 5. Conclusions

In conclusion, this analysis suggests that laboratory assessments prior to each dose of nivolumab or nivolumab+ipilimumab are not mandatory in clinical practice in patients with mRCC. Reducing the frequency of testing did not substantially increase the risk of missing events relevant to treatment decisions, particularly when using the disease-specific dose delay and discontinuation criteria defined in the TITAN-RCC protocol. Applying these, in asymptomatic patients our analysis suggests that the chosen frequency of laboratory testing can be considered sufficient, offering potential time and resource savings in clinical settings. Careful consideration should be given to which laboratory values need to be determined, also in relation to the presence or absence of symptoms. Nevertheless, a re-increase of frequencies could be considered if dose delay criteria were met at least once, especially for lipase and creatinine.

## Figures and Tables

**Table 1:** Dose delay and discontinuation criteria according to the TITAN-RCC protocol or to the SmPC of nivolumab.

Lab Value	Dose Delay Criteria		Discontinuation Criteria	
	Protocol of TITAN-RCC	Current SmPC	Protocol of TITAN-RCC	Current SmPC
**Leukocytes** (leukocytosis)	**grade 3** (>100,000/mm^3^)	**grade 3** (>100,000/mm^3^), **first occurrence**	**grade 4** (clinical manifestations of leukostasis; urgent intervention indicated)	**grade 4** (clinical manifestations of leukostasis; urgent intervention indicated) or **recurrent grade 3** (>100,000/mm^3^) ^1,2^
**ASAT or ** **ALAT**	**baseline normal: ** **grade 2 or 3** (>3.0 × ULN) ^3^ **baseline abnormal:** **grade 3** (>5.0 × ULN) ^3^	**grade 2** (>3.0–5.0 × ULN)	≥8 × ULN or AST/ALT ≥ 3 × ULN *and* bilirubin ≥2 × ULN	**grade 3 or 4** (>5.0 × ULN)
**Bilirubin**	**baseline normal:** **grade 2 or 3** (>1.5 × ULN) ^3^ **baseline abnormal:** **grade 3** (>3.0 × ULN) ^3^	**grade 2** (>1.5–3.0 × ULN)	≥5 × ULN or AST/ALT ≥ 3 × ULN *and* bilirubin ≥2 × ULN	**grade 3 or 4** (>3.0 × ULN)
**Creatinine**	**grade 3** (>3.0xbaseline or >3.0–6.0 × ULN)	**grade 2** (>1.5–3.0xbaseline or >1.5–3.0 × ULN) or **grade 3** (>3.0xbaseline or >3.0–6.0 × ULN)	**grade 4** (>6.0 × ULN)	**grade 4** (>6.0 × ULN)
**Amylase or Lipase**	**grade 3** (>2.0–5.0 × ULN) (**symptomatic** ^4^)	**grade 3** (>2.0–5.0 × ULN), **first occurrence**	**grade 4** (>5.0 × ULN) (**symptomatic** ^4^)	**grade 4** (>5.0 x ULN) or **recurrent grade 3** (>2.0–5.0 × ULN) ^1,2^
**TSH ** (hypothyroidism)	**grade 3** (severe symptoms; limiting self care ADL; hospitalisation indicated)	**grade 2** (symptomatic; thyroid replacement indicated ^5^; limiting instrumental ADL) or **grade 3** (severe symptoms; limiting self care ADL; hospitalisation indicated)	**grade 4** (life-threatening consequences; urgent intervention indicated)	**grade 4** (life-threatening consequences; urgent intervention indicated)

ADL, activities of daily life; ALAT, alanine aminotransferase; ASAT, aspartate aminotransferase; CTCAE, Common Terminology Criteria of Adverse Events; SmPC, summary of product characteristics/product information; TSH, thyroid-stimulating hormone; ULN, upper limit of normal; LLN, lower limit of normal. ^1^ Per SmPC for nivolumab discontinuation is also recommended for persistent grade 2 or 3 despite treatment modification (dose delay). This is not the subject of our analyses, since we assessed monitoring of laboratory values on treatment. ^2^ Per SmPC for nivolumab discontinuation is also recommended for the inability to reduce corticosteroid dose to 10 mg prednisone or equivalent per day. This criterion has not been applied since it is not dependent on laboratory analyses and therewith not the subject of our analyses. ^3^ Until discontinuation criterion is met. ^4^ Defined per protocol of TITAN-RCC, independent of CTCAE v4.0. ^5^ Only first occurrence of an indication for thyroid replacement was counted for usually long-term or permanent substitution.

**Table 2:** Simulated assessment of laboratory values.

	Simulated Assessment of Laboratory Values	
	Variant A	Variant B (Control; Supplementary Materials)
**Nivolumab induction** **monotherapy**	First and second visit, every second visit thereafter	First visit, every second visit thereafter
**Nivolumab+ipilimumab boost phase**	First, second, and fourth visit	First and third visit
**Nivolumab maintenance monotherapy ^(1)^**	First and second visit, every second visit thereafter	First visit, every second visit thereafter
**Follow-up**	No assessment	No assessment

^(1)^ Either after nivolumab induction or after nivolumab+ipilimumab boosts.

**Table 3:** Baseline characteristics.

	Total (n = 207)
**Median age, years (range, IQR)**	65 (20–87, 57–71)
**Line of therapy**	
**First-line**	109 (53)
**Second-line**	98 (47)
**Sex, n (%)**	
**Male**	147 (71)
**Female**	60 (29)
**IMDC risk factors, n (%)**	
**Karnofsky Performance Status < 80%**	34 (16)
**Initial diagnosis ≤ 12 months**	135 (65)
**Haemoglobin < LLN**	110 (53)
**Platelet count > ULN**	39 (19)
**Neutrophil count > ULN**	26 (13)
**Calcium (corrected) > ULN**	27 (13)
**IMDC risk group, n (%)**	
**Favourable**	9 (4)
**Intermediate**	147 (71)
**Poor**	51 (25)

IMDC, International Metastatic Renal Cell Carcinoma Database Consortium; IQR, interquartile range; LLN, lower limit of normal; ULN, upper limit of normal.

**Table 4:** Missed elevation of CTCAE grade by at least one grade.

	Missed Elevation per Patient, n/N (%)	Missed Elevation per Assessment, n/N (%)
**Leukocytes**	32/207 (15)	47/5487 (0.9)
**ALAT**	42/207 (20)	73/5396 (1)
**ASAT**	44/207 (21)	54/5252 (1)
**Bilirubin**	20/207 (10)	25/5404 (0.5)
**Creatinine**	83/207 (40)	192/5475 (4)
**Amylase**	52/207 (25)	120/4606 (3)
**Lipase**	66/207 (32)	152/4860 (3)
**TSH**	51/207 (25)	100/5297 (2)

Data were obtained with variant A. For variant B and *p* values for the per-patient level see Supplementary Table S1. ALAT, alanine aminotransferase; ASAT, aspartate aminotransferase; TSH, thyroid-stimulating hormone.

**Table 5:** Missed dose delay and discontinuation criteria per assessment and per patient, criteria defined according to the TITAN-RCC protocol.

	Per Patient, n/N (%)		Per Assessment, n/N (%)	
	Dose Delay	Discontinuation	Dose Delay	Discontinuation
**Nivolumab monotherapy (induction and maintenance)**				
**Leukocytes**	1/207 (0.5)	0/207 (0)	1/4522 (<0.1)	0/4522 (0)
**ALAT**	1/207 (0.5)	0/207 (0)	1/4447 (<0.1)	0/4447 (0)
**ASAT**	1/207 (0.5)	0/207 (0)	2/4327 (<0.1)	0/4327 (0)
**Bilirubin**	1/207 (0.5)	0/207 (0)	1/4458 (<0.1)	0/4458 (0)
**Creatinine**	0/207 (0)	0/207 (0)	0/4511 (0)	0/4511 (0)
**Amylase**	2/207 (1)	1/207 (0.5)	2/3965 (<0.1)	1/3965 (<0.1)
**Lipase**	15/207 (7)	4/207 (2)	25/4204 (0.6)	5/4204 (0.1)
**TSH**	2/207 (1)	0/207 (0)	3/4382 (<0.1)	0/4382 (0)
**Nivolumab+ipilimumab boost**				
**Leukocytes**	0/138 (0)	0/138 (0)	0/518 (0)	0/518 (0)
**ALAT**	0/138 (0)	0/138 (0)	0/510 (0)	0/510 (0)
**ASAT**	0/137 (0)	0/137 (0)	0/502 (0)	0/502 (0)
**Bilirubin**	1/137 (0.7)	0/137 (0)	1/510 (0.2)	0/510 (0)
**Creatinine**	0/138 (0)	0/138 (0)	0/518 (0)	0/518 (0)
**Amylase**	1/132 (0.8)	0/132 (0)	1/465 (0.2)	0/465 (0)
**Lipase**	4/134 (3)	1/134 (0.7)	4/480 (0.8)	1/480 (0.2)
**TSH**	0/138 (0)	0/138 (0)	0/509 (0)	0/509 (0)

Data were obtained with variant A. For variant B and *p* values for the per-patient level see Supplementary Table S2. *Yellow*: dose delay or discontinuation criteria would have been missed at least once on a per-assessment or per-patient level. ALAT, alanine aminotransferase; ASAT, aspartate aminotransferase; TSH, thyroid-stimulating hormone.

**Table 6:** Missed dose delay and discontinuation criteria per assessment and per patient, criteria defined according to the SmPC of nivolumab.

	Per Patient, n/N (%)		Per Assessment, n/N (%)	
	Dose Delay	Discontinuation	Dose Delay	Discontinuation
**Nivolumab monotherapy (induction and maintenance)**				
**Leukocytes**	1/207 (0.5)	0/207 (0)	1/4522 (<0.1)	0/4522 (0)
**ALAT**	1/207 (0.5)	0/207 (0)	1/4447 (<0.1)	0/4447 (0)
**ASAT**	3/207 (1.4)	0/207 (0)	6/4327 (0.1)	0/4327 (0)
**Bilirubin**	1/207 (0.5)	0/207 (0)	4/4458 (<0.1)	0/4458 (0)
**Creatinine**	19/207 (9)	0/207 (0)	134/4511 (3)	0/4511 (0)
**Amylase**	0/207 (0)	1/207 (0.5)	0/3965 (0)	1/3965 (<0.1)
**Lipase**	7/207 (3)	7/207 (3)	7/4204 (0.1)	14/4204 (0.3)
**TSH**	10/207 (0.5)	0/207 (0)	10/4382 (0.2)	0/4382 (0)
**Nivolumab+ipilimumab boost**				
**Leukocytes**	0/138 (0)	0/138 (0)	0/518 (0)	0/518 (0)
**ALAT**	0/138 (0)	0/138 (0)	0/510 (0)	0/510 (0)
**ASAT**	0/137 (0)	0/137 (0)	0/502 (0)	0/502 (0)
**Bilirubin**	0/137 (0)	0/137 (0)	0/510 (0)	0/510 (0)
**Creatinine**	6/138 (4)	0/138 (0)	9/518 (2)	0/518 (0)
**Amylase**	0/132 (0)	0/132 (0)	0/465 (0)	0/465 (0)
**Lipase**	2/134 (1)	2/134 (1)	2/480 (0.4)	2/480 (0.4)
**TSH**	1/138 (0)	0/138 (0)	1/509 (0.2)	0/509 (0)

Data were obtained with variant A. For variant B and *p* values for the per-patient level see Supplementary Table S3. *Orange:* lower incidence as compared to Table 5 (criteria defined according to the TITAN-RCC protocol); *green:* higher incidence as compared to Table 5. ALAT, alanine aminotransferase; ASAT, aspartate aminotransferase; TSH, thyroid-stimulating hormone.

## Data Availability

The data presented in this study are available on request from the corresponding author.

## Supplementary Materials

The following supporting information can be downloaded at: https://www.mdpi.com/article/10.3390/cancers16122287/s1, Table S1. Missed elevation of CTCAE-grade by at least one grade; Table S2. Missed dose delay and discontinuation criteria per assessment and per patient, criteria defined according to the TITAN-RCC protocol (data obtained with variant B); Table S3. Missed dose delay and discontinuation criteria per assessment and per patient, criteria defined according to the SmPC of nivolumab (data obtained with variant B).
