# Supplementary material for: Reasonability of Frequent Laboratory Analyses during Therapy with Nivolumab and Nivolumab+Ipilimumab in Patients with Advanced or Metastatic Renal Cell Carcinoma during the Phase 2 Clinical Trial TITAN-RCC
Source: Cancers (Basel). 2024 Jun 20;16(12):2287. doi: 10.3390/cancers16122287 (PMC11201947; doi:10.3390/cancers16122287)
Supplement: Supplementary file 1 [file cancers-16-02287-s001.zip › cancers-2982201-supplementary.pdf]

Supplementary Data

Supplementary Table S1. Missed elevation of CTCAE-grade by at least one grade.

	Missed elevation per patient, n/N (%) [1)]	*p* value [2)]	Missed elevation per assessment, n/N (%) [1)]
**Leukocytes**	32/207 (15)	0.588	54/5487 (1)
**ALAT**	56/207 (27)	0.151	94/5396 (2)
**ASAT**	38/207 (18)	0.526	58/5252 (1)
**Bilirubin**	16/207 (8)	0.344	28/5404 (0.5)
**Creatinine**	86/207 (42)	0.749	186/5475 (3)
**Amylase**	60/207 (29)	0.268	110/4606 (2)
**Lipase**	77/207 (37)	0.061	173/4860 (4)
**TSH**	58/207 (28)	0.167	112/5297 (2)

[1)] Data were obtained with variant B. [2)] p values for the per-patient level were calculated using McNemar's test and compare to variant A (table 4 in main manuscript).
ALAT, alanine aminotransferase; ASAT, aspartate aminotransferase; TSH, thyroid-stimulating hormone

Supplementary Table S2. Missed dose delay and discontinuation criteria per assessment and per patient, criteria defined according to the TITAN-RCC protocol.

	Per patient, n/N (%)				Per assessment, n/N (%)	
	Dose delay [1)]	*p* value [2)]	Discontinu-ation [1)]	*p* value [2)]	Dose delay [1)]	Discontinuation [1)]
**Nivolumab monotherapy (induction and maintenance)**						
**Leukocytes**	0/207 (0)	n.a.	0/207 (0)	n.a.	0/4522 (0)	0/4522 (0)
**ALAT**	3/207 (1)	0.625	0/207 (0)	n.a.	3/4447 (<0.1)	0/4447 (0)
**ASAT**	1/207 (0.5)	1.00	0/207 (0)	n.a.	1/4327 (<0.1)	0/4327 (0)
**Bilirubin**	0/207 (0)	n.a.	0/207 (0)	n.a.	0/4458 (0)	0/4458 (0)
**Creatinine**	1/207 (0.5)	n.a.	0/207 (0)	n.a.	1/4511 (<0.1)	0/4511 (0)
**Amylase**	3/207 (1)	0.500	1/207 (0.5)	1.000	3/3965 (<0.1)	1/3965 (<0.1)
**Lipase**	17/207 (8)	0.774	7/207 (3)	0.250	27/4204 (0.6)	7/4204 (0.2)
**TSH**	1/207 (0.5)	1.000	0/207 (0)	n.a.	1/4382 (<0.1)	0/4382 (0)
**Nivolumab+ipilimumab boost**						
**Leukocytes**	0/138 (0)	n.a.	0/138 (0)	n.a.	0/518 (0)	0/518 (0)
**ALAT**	0/138 (0)	n.a.	0/138 (0)	n.a.	0/510 (0)	0/510 (0)
**ASAT**	0/137 (0)	n.a.	0/137 (0)	n.a.	0/502 (0)	0/502 (0)
**Bilirubin**	0/137 (0)	n.a.	0/137 (0)	n.a.	0/510 (0)	0/510 (0)
**Creatinine**	1/138 (0.7)	n.a.	0/138 (0)	n.a.	1/518 (0.2)	0/518 (0)
**Amylase**	1/132 (0.8)	1.000	0/132 (0)	n.a.	1/465 (0.2)	0/465 (0)
**Lipase**	4/134 (3)	1.000	1/134 (0.7)	1.000	4/480 (0.8)	1/480 (0.2)
**TSH**	0/138 (0)	n.a.	0/138 (0)	n.a.	0/509 (0)	0/509 (0)

[1)] Data were obtained with variant B. [2)] p values for the per-patient level were calculated using McNemar's test and compare to variant A (table 5 in main manuscript).

Yellow: dose delay or discontinuation criteria would have been missed at least once on a per-assessment or per-patient level.

ALAT, alanine aminotransferase; ASAT, aspartate aminotransferase; TSH, thyroid-stimulating hormone

Supplementary Table S3. Missed dose delay and discontinuation criteria per assessment and per patient, criteria defined according to the SmPC of nivolumab.

	Per patient, n/N (%)				Per assessment, n/N (%)	
	Dose delay [1)]	*p* value [2)]	Discontinu-ation [1)]	*p* value [2)]	Dose delay [1)]	Discontinuation [1)]
**Nivolumab monotherapy (induction and maintenance)**						
**Leukocytes**	0/207 (0)	n.a.	0/207 (0)	n.a.	0/4522 (0)	0/4522 (0)
**ALAT**	2/207 (1)	1.000	1/207 (0.5)	n.a.	2/4447 (<0.1)	1/4447 (<0.1)
**ASAT**	4/207 (2)	1.000	0/207 (0)	n.a.	9/4327 (0.2)	0/4327 (0)
**Bilirubin**	2/207 (1)	1.000	0/207 (0)	n.a.	4/4458 (<0.1)	0/4458 (0)
**Creatinine**	22/207 (11)	0.453	0/207 (0)	n.a.	144/4511 (3)	0/4511 (0)
**Amylase**	0/207 (0)	n.a.	1/207 (0.5)	1.000	0/3965 (0)	1/3965 (<0.1)
**Lipase**	9/207 (4)	0.344	11/207 (5)	0.125	9/4204 (0.2)	19/4204 (0.4)
**TSH**	6/207 (0)	0.063	0/207 (0)	n.a.	6/4382 (0.1)	0/4382 (0)
**Nivolumab+ipilimumab boost**						
**Leukocytes**	0/138 (0)	n.a.	0/138 (0)	n.a.	0/518 (0)	0/518 (0)
**ALAT**	0/138 (0)	n.a.	0/138 (0)	n.a.	0/510 (0)	0/510 (0)
**ASAT**	0/137 (0)	n.a.	0/137 (0)	n.a.	0/502 (0)	0/502 (0)
**Bilirubin**	0/137 (0)	n.a.	0/137 (0)	n.a.	0/510 (0)	0/510 (0)
**Creatinine**	6/138 (4)	1.00	0/138 (0)	n.a.	9/518 (2)	0/518 (0)
**Amylase**	0/132 (0)	n.a.	0/132 (0)	n.a.	0/465 (0)	0/465 (0)
**Lipase**	2/134 (1)	1.000	2/134 (1)	1.000	2/480 (0.4)	2/480 (0.4)
**TSH**	1/138 (0)	1.000	0/138 (0)	n.a.	1/509 (0.2)	0/509 (0)

[1)] Data were obtained with variant B. [2)] p values for the per-patient level were calculated using McNemar's test and compare to variant A (table 6 in main manuscript).

Orange: lower incidence as compared to supplementary table 2 (criteria defined according to the TITAN-RCC protocol);

Green: higher incidence as compared to supplementary table 2.

ALAT, alanine aminotransferase; ASAT, aspartate aminotransferase; TSH, thyroid-stimulating hormone
